# Supplementary figures and images for: Scavenger receptor endocytosis controls apical membrane morphogenesis in the Drosophila airways
Source: eLife. 2023 Sep 14;12:e84974. doi: 10.7554/eLife.84974 (PMC10564452; doi:10.7554/eLife.84974)

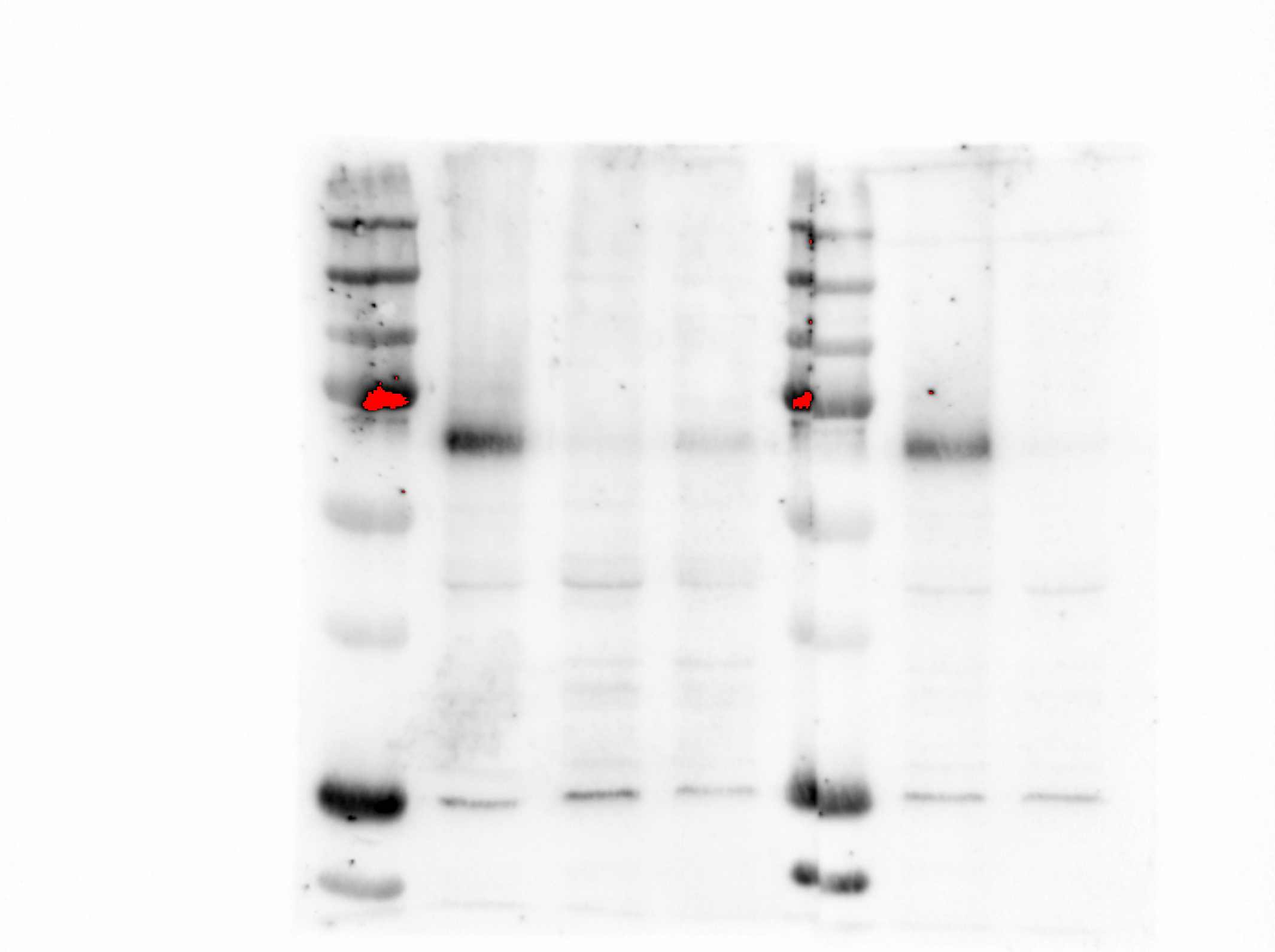

Supplement: Figure 1—figure supplement 1—source data 1. [file elife-84974-fig1-figsupp1-data1.zip › Figure 1ΓÇöfigure supplement 1- source data 1/raw-emp_ w1118,empMut,df_emp 1-500, 1-1000_10_last blot.tif]

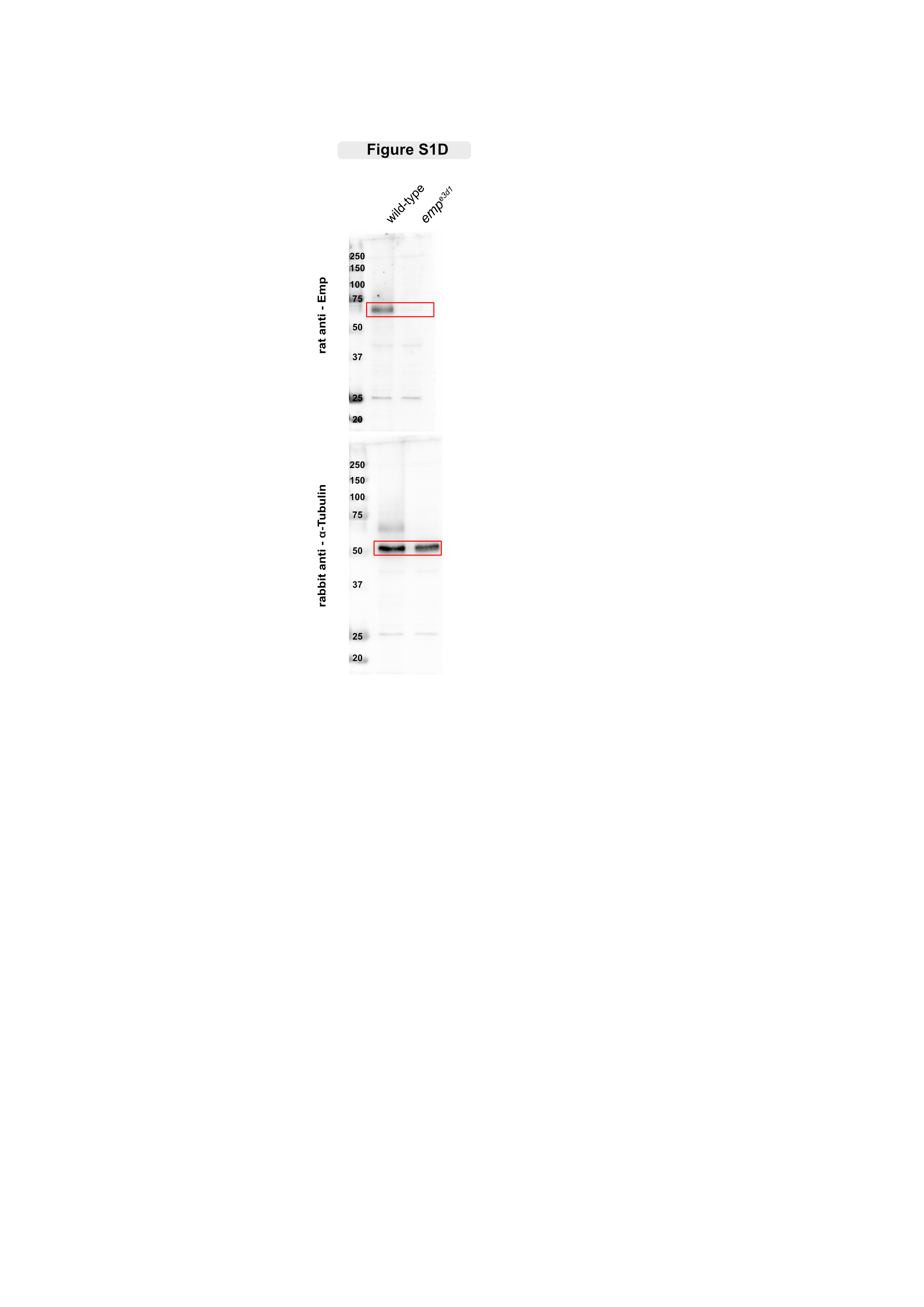

Supplement: Figure 1—figure supplement 1—source data 1. [file elife-84974-fig1-figsupp1-data1.zip › Figure 1ΓÇöfigure supplement 1- source data 1/Figure supl 1D.tiff]

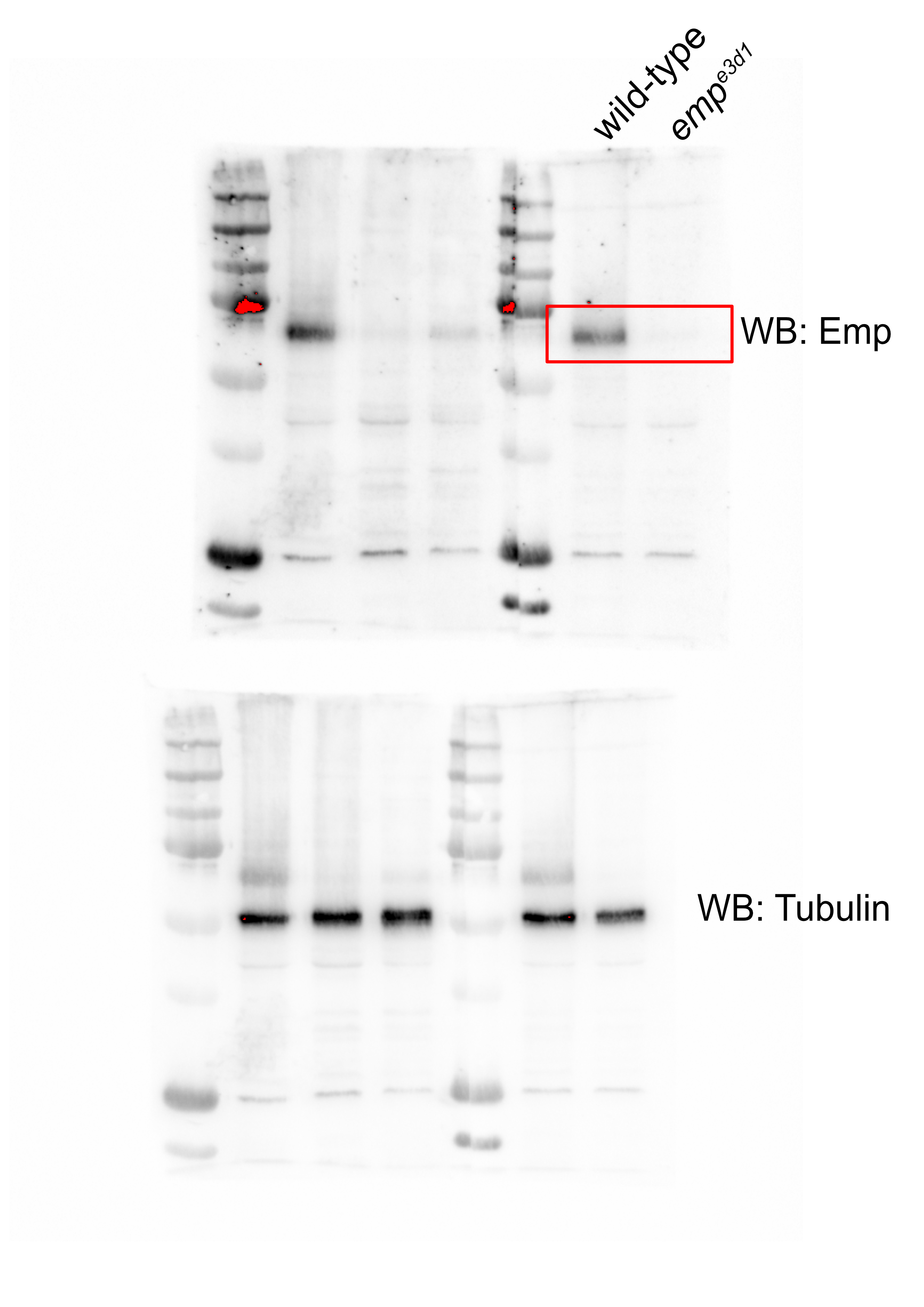

Supplement: Figure 1—figure supplement 1—source data 1. [file elife-84974-fig1-figsupp1-data1.zip › Figure 1ΓÇöfigure supplement 1- source data 1/Raw and Labels.tiff]

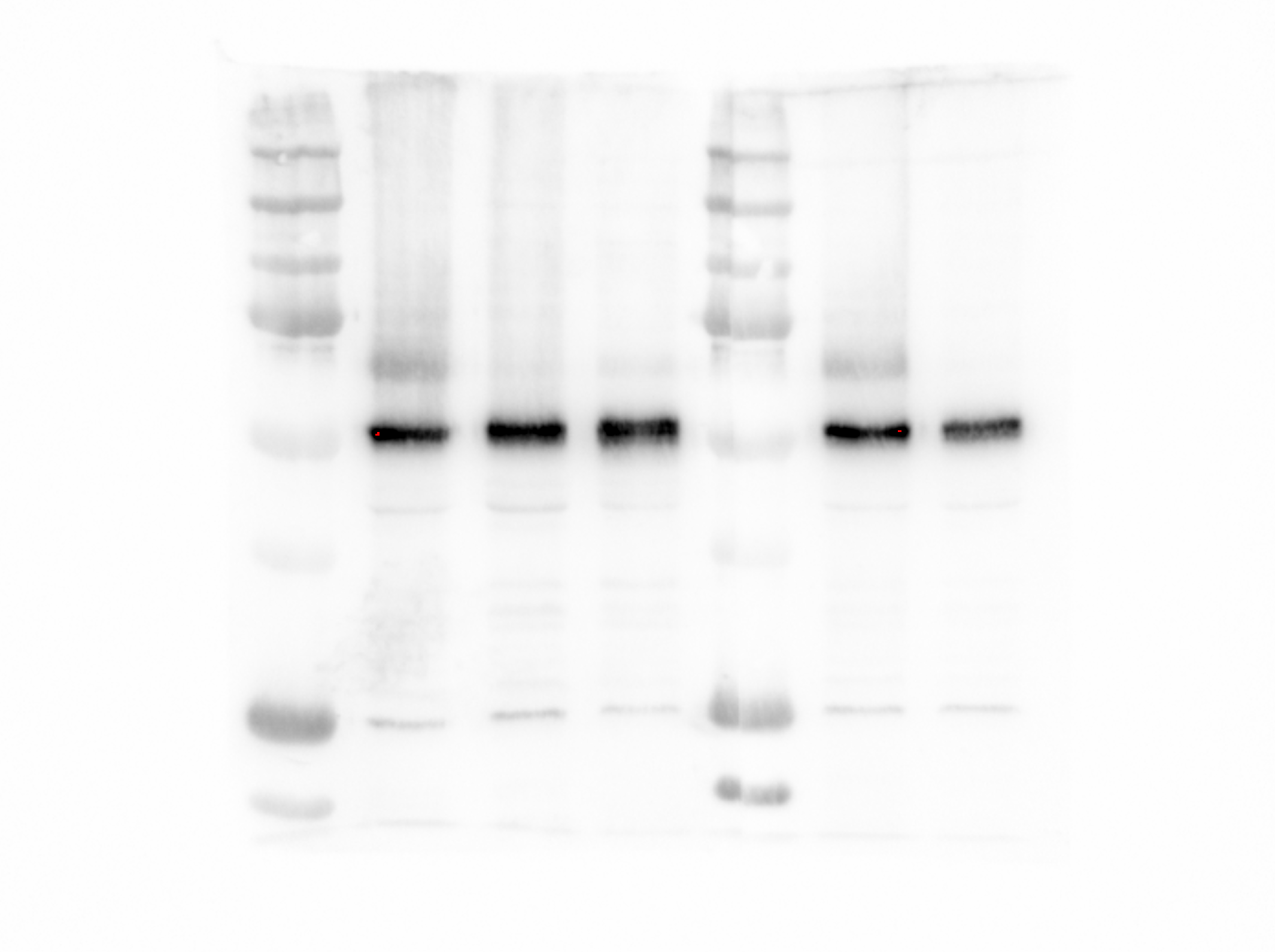

Supplement: Figure 1—figure supplement 1—source data 1. [file elife-84974-fig1-figsupp1-data1.zip › Figure 1ΓÇöfigure supplement 1- source data 1/raw-emp_ w1118,empMut,df_emp 1-500, 1-1000_(a-tub)2.3sec_last blot.tif]

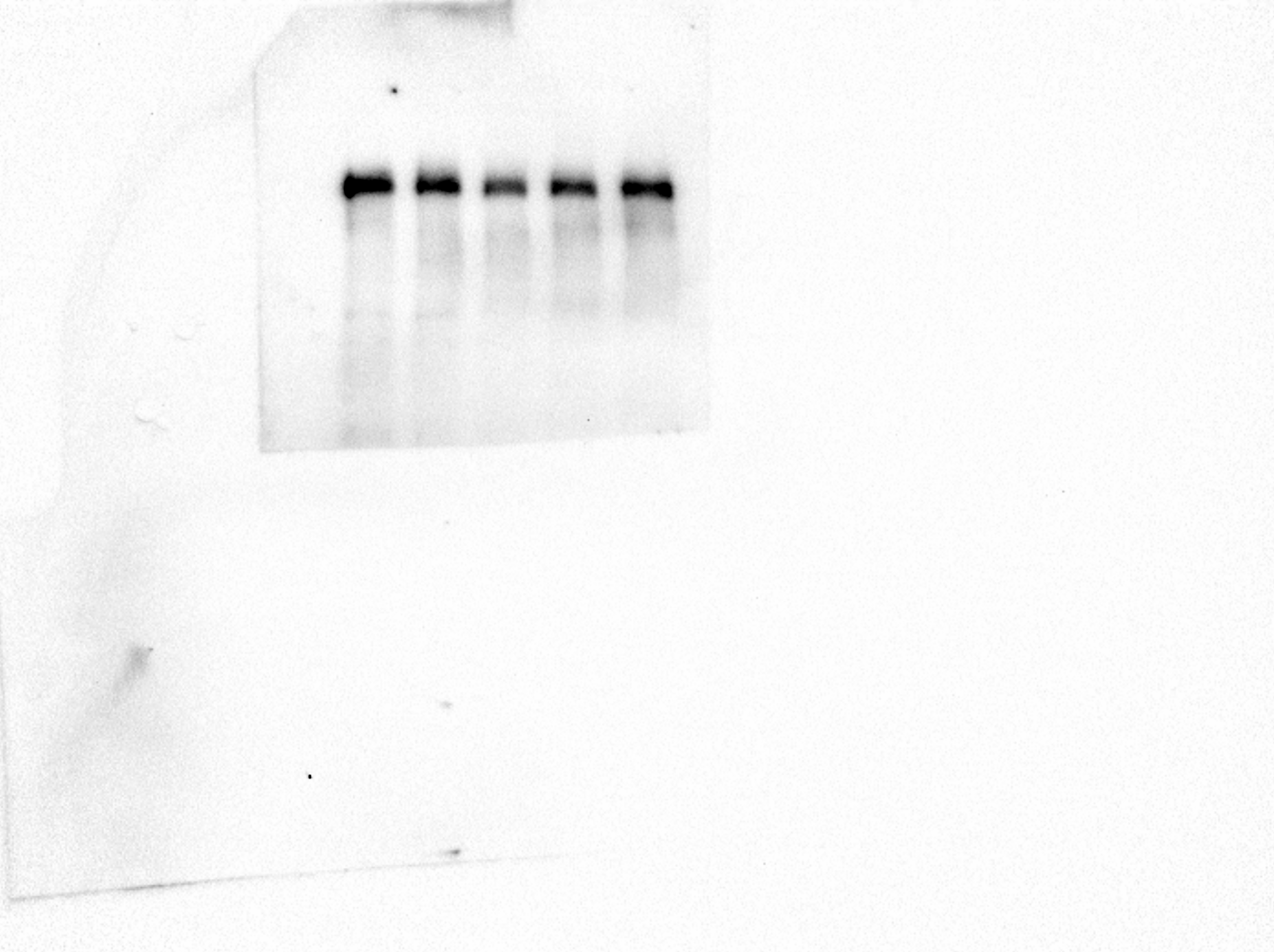

Supplement: Figure 4—source data 1. [file elife-84974-fig4-data1.zip › Figure 4ΓÇösource data 1/raw Input_ab-Crb_17sec2.tif]

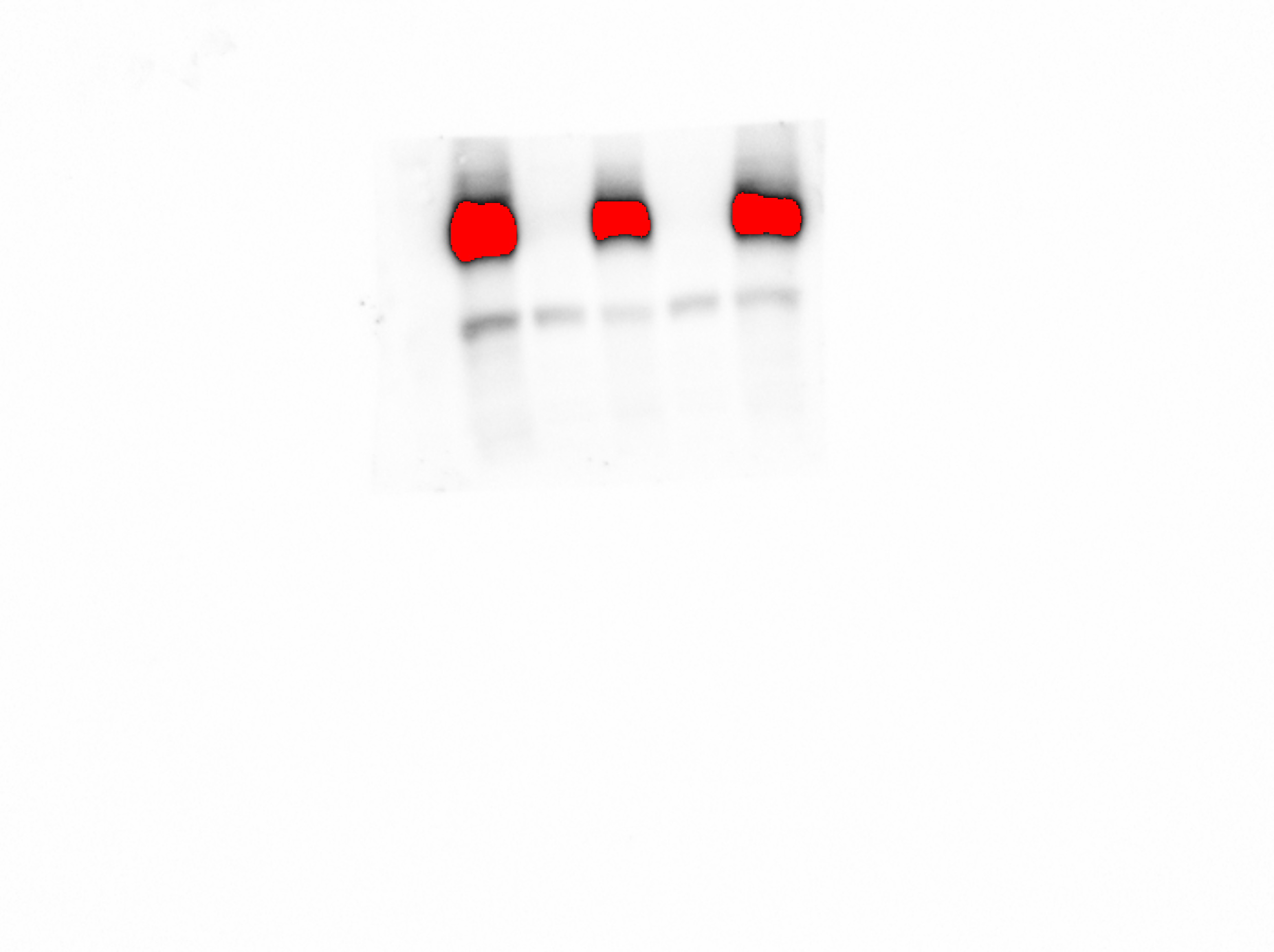

Supplement: Figure 4—source data 1. [file elife-84974-fig4-data1.zip › Figure 4ΓÇösource data 1/raw Input_ab-Tub_2sec.tif]

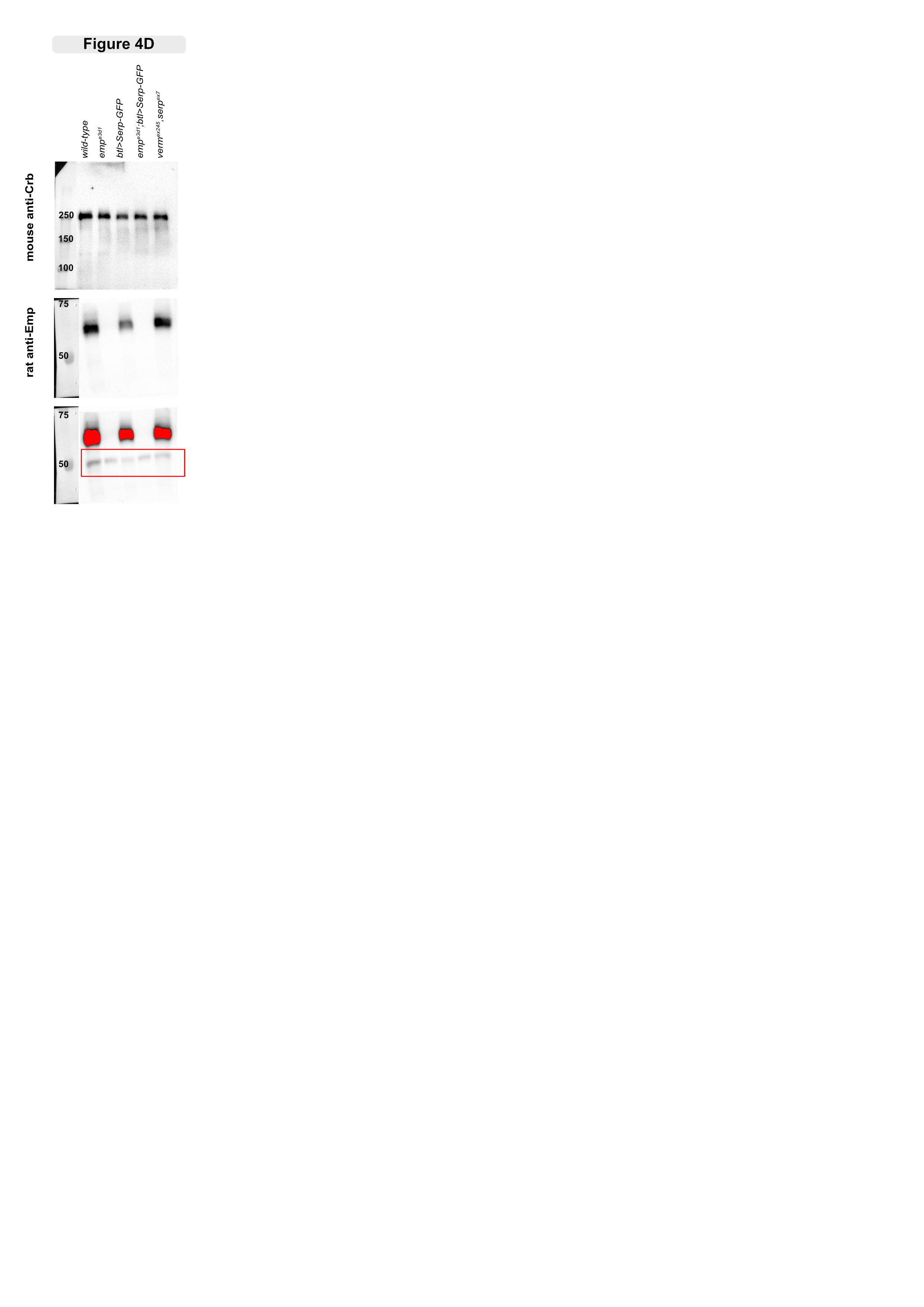

Supplement: Figure 4—source data 1. [file elife-84974-fig4-data1.zip › Figure 4ΓÇösource data 1/Figure 4D.tiff]

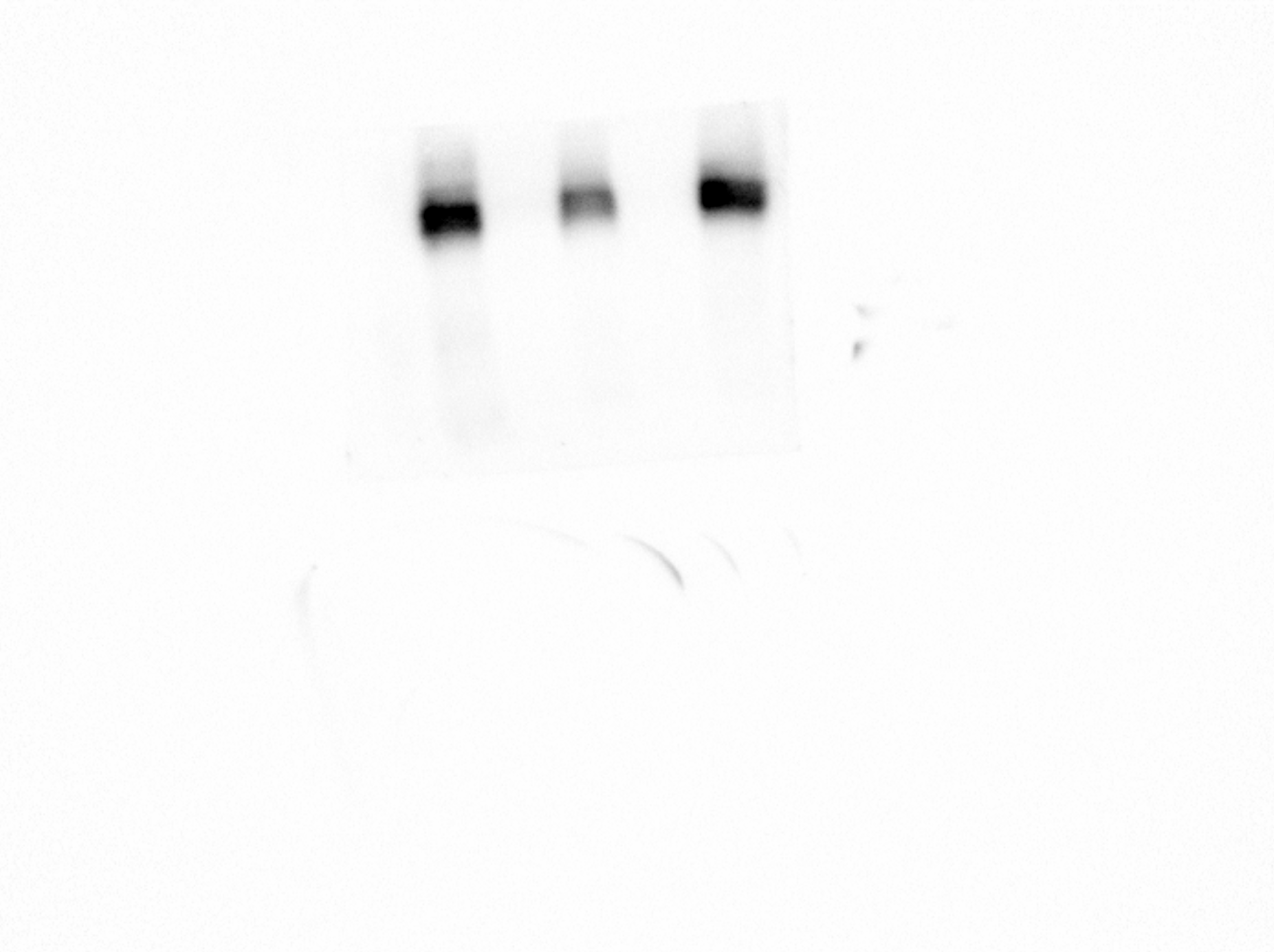

Supplement: Figure 4—source data 1. [file elife-84974-fig4-data1.zip › Figure 4ΓÇösource data 1/raw Input_ab-Emp_0,3sec.tif]

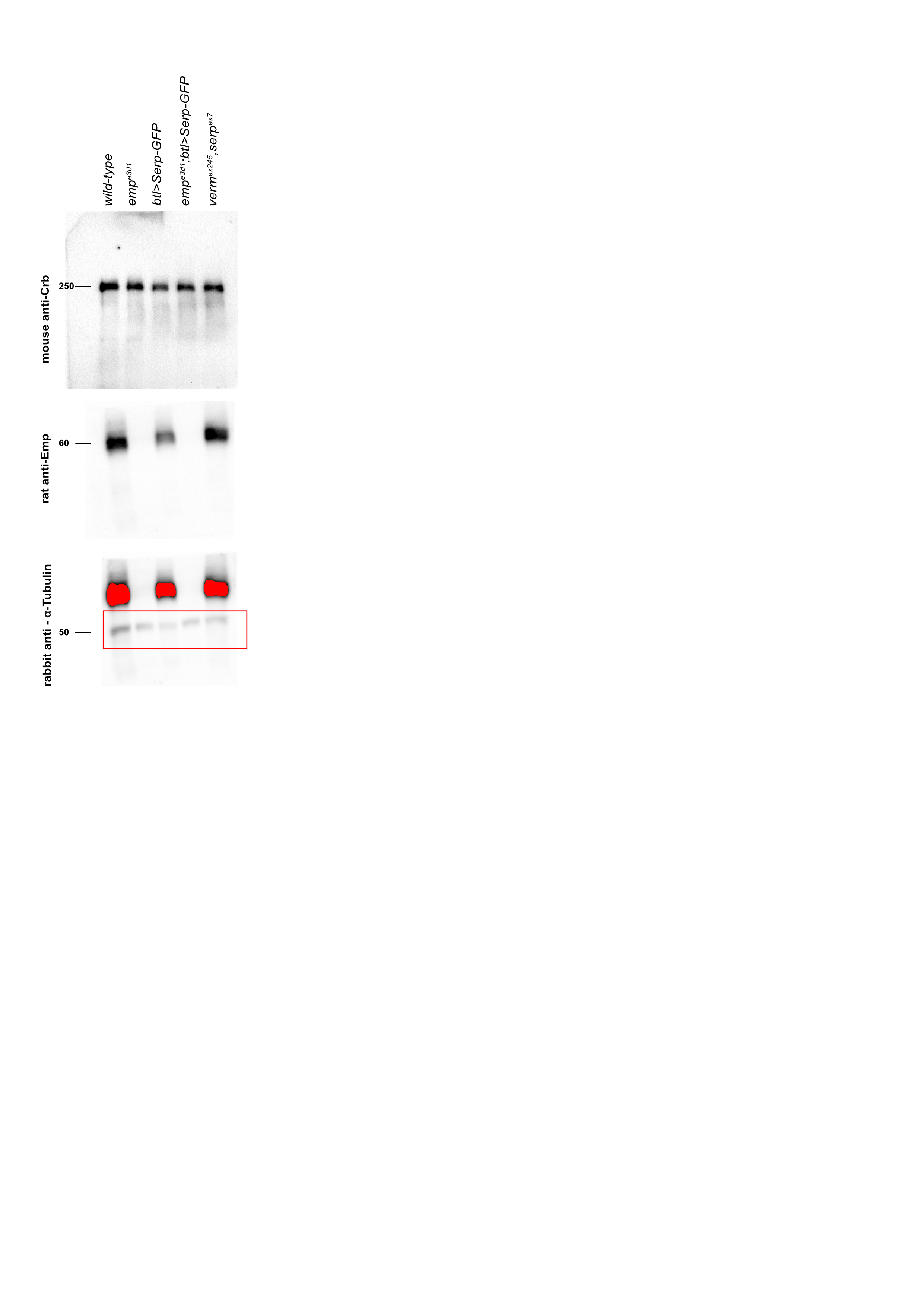

Supplement: Figure 4—source data 1. [file elife-84974-fig4-data1.zip › Figure 4ΓÇösource data 1/raw and labels.tiff]

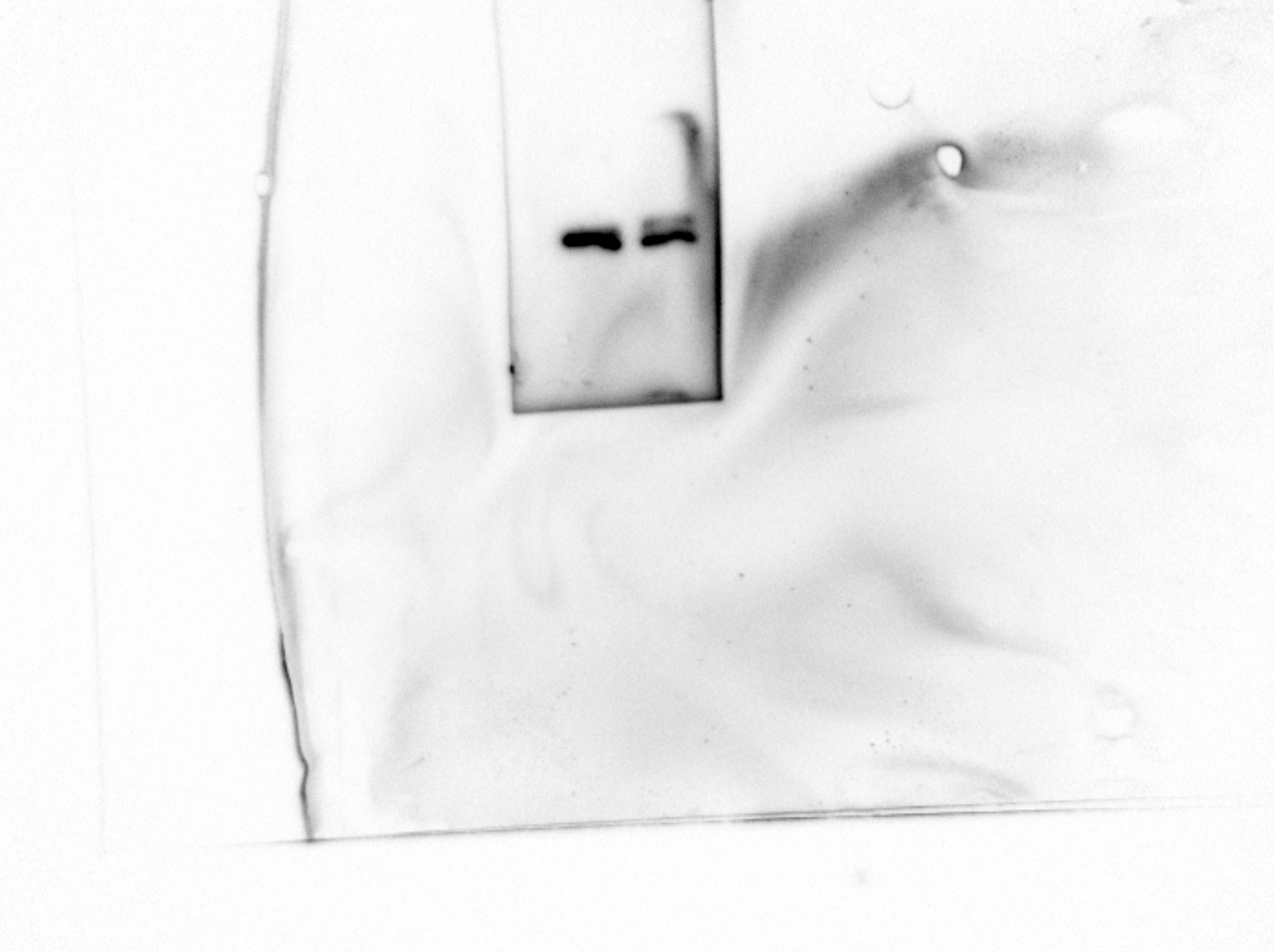

Supplement: Figure 5—source data 1. [file elife-84974-fig5-data1.zip › Figure 5ΓÇösource data 1/raw Input_ab-De-cad_0,5sec.tif]

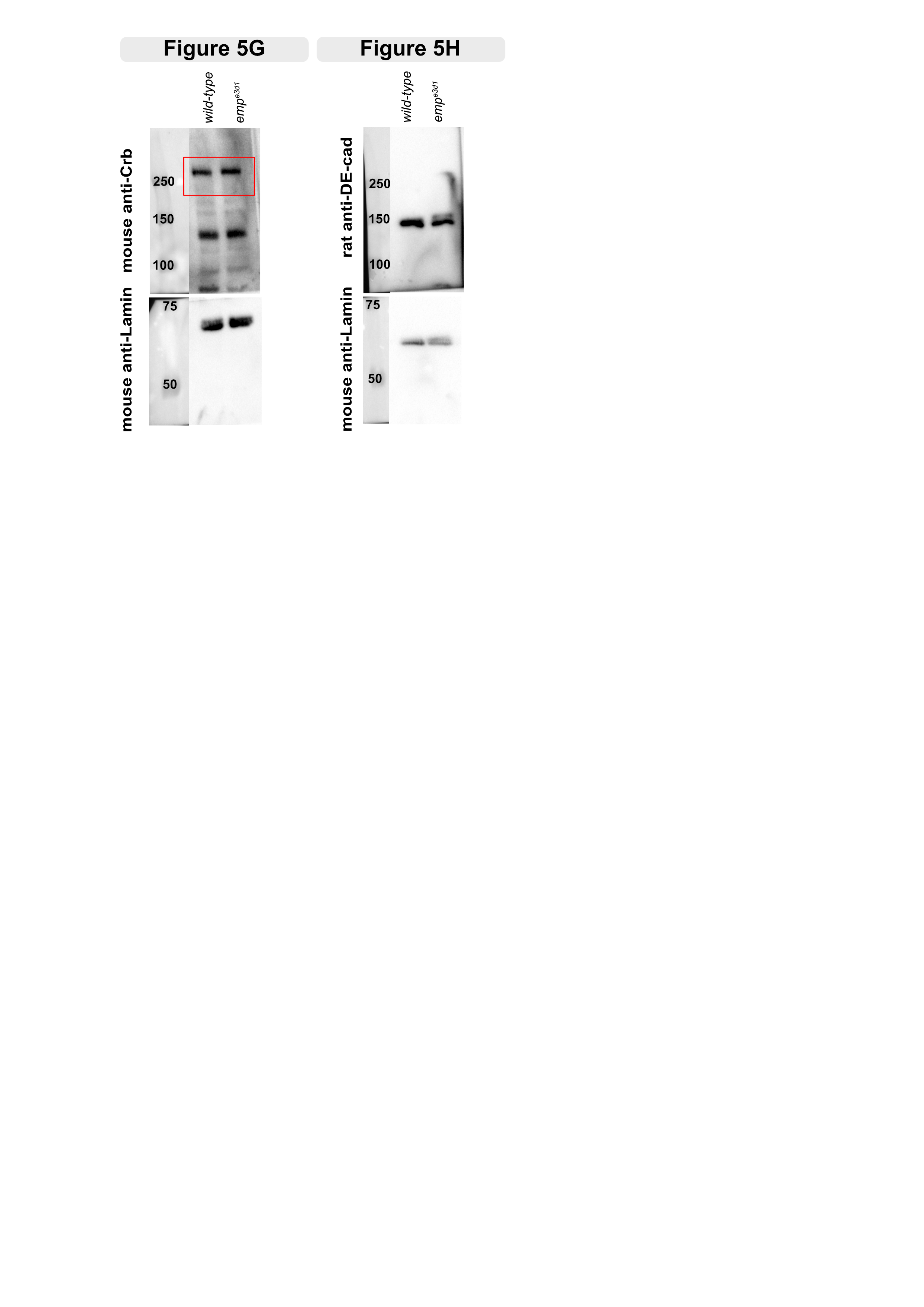

Supplement: Figure 5—source data 1. [file elife-84974-fig5-data1.zip › Figure 5ΓÇösource data 1/Figure 5G,H.tiff]

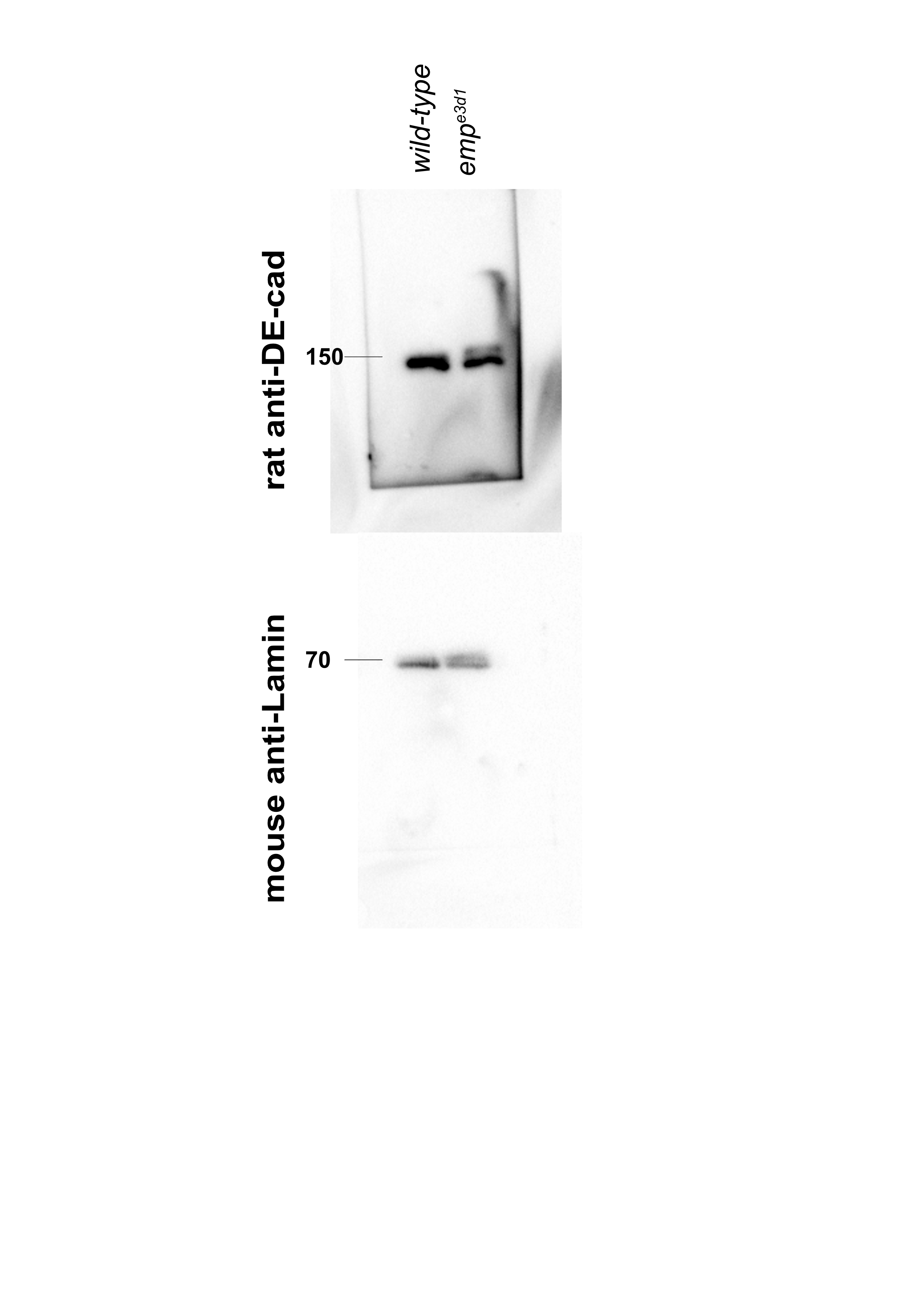

Supplement: Figure 5—source data 1. [file elife-84974-fig5-data1.zip › Figure 5ΓÇösource data 1/raw and labels 5H.tiff]

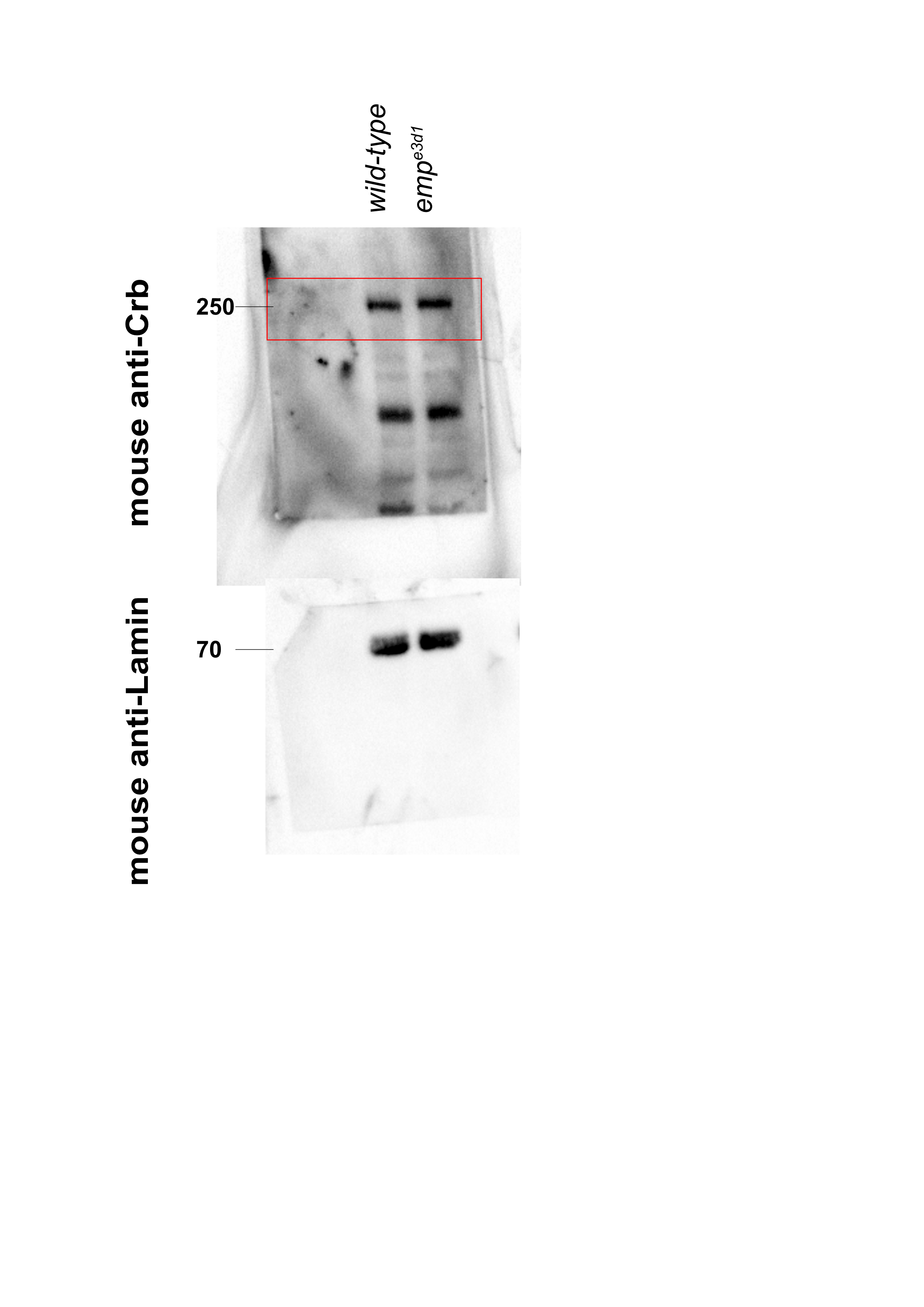

Supplement: Figure 5—source data 1. [file elife-84974-fig5-data1.zip › Figure 5ΓÇösource data 1/raw and labels 5G.tiff]

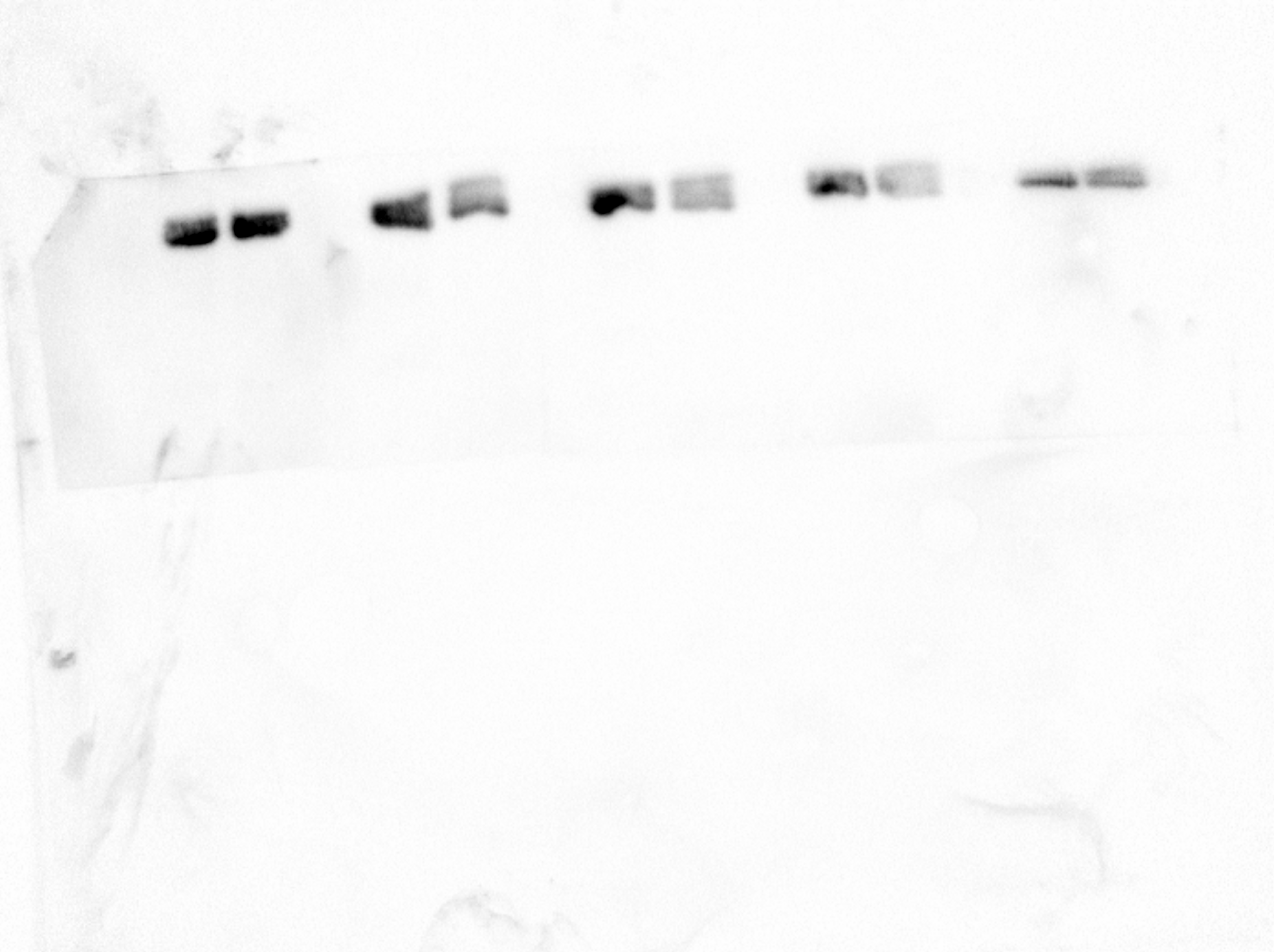

Supplement: Figure 5—source data 1. [file elife-84974-fig5-data1.zip › Figure 5ΓÇösource data 1/raw Input_ab-lamin_0,5sec.tif]

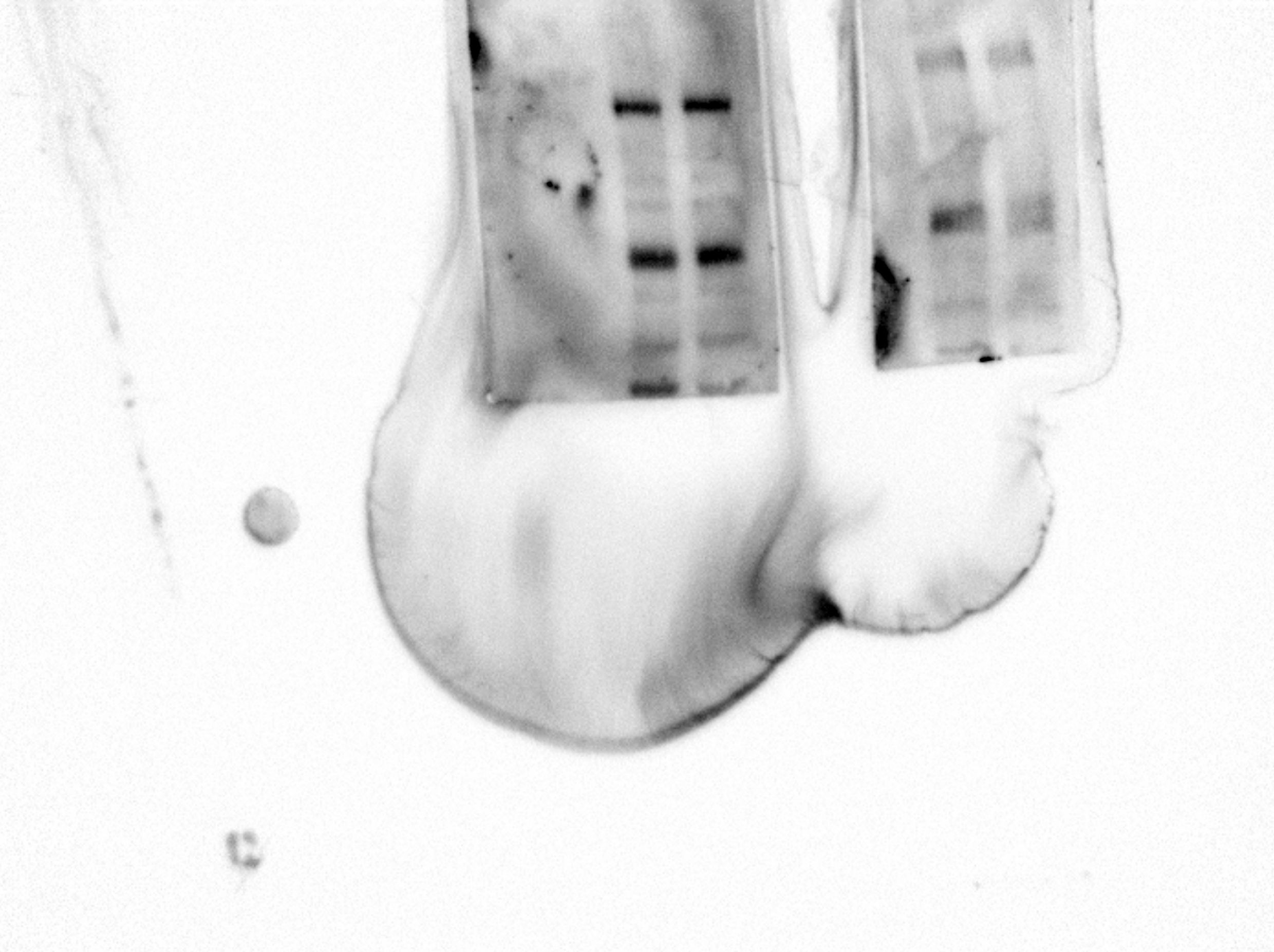

Supplement: Figure 5—source data 1. [file elife-84974-fig5-data1.zip › Figure 5ΓÇösource data 1/raw Input_ab-crb_6,2sec.tif]

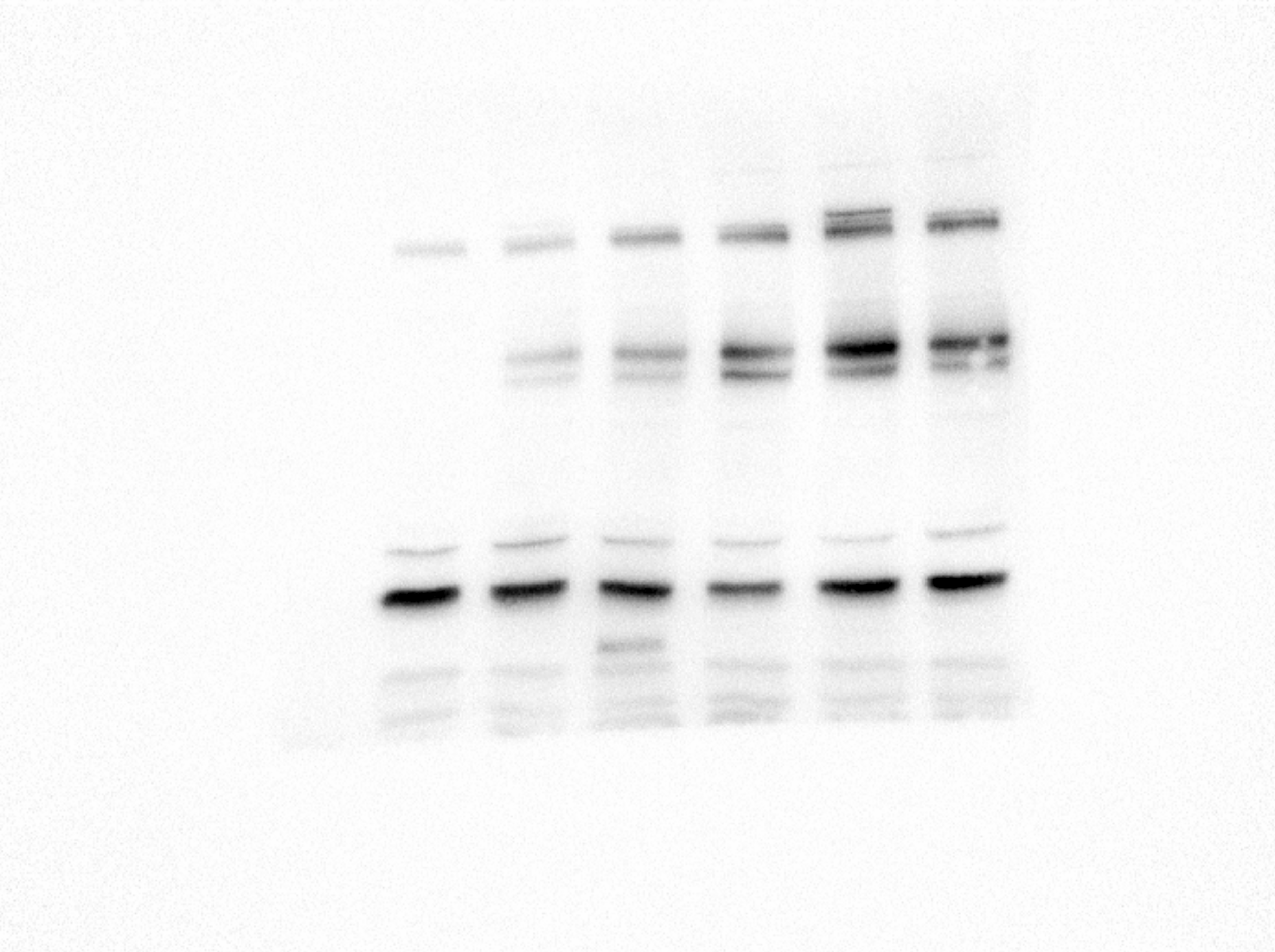

Supplement: Figure 6—source data 1. [file elife-84974-fig6-data1.zip › Figure 6ΓÇösource data 1/raw Input_ab-tub_0,5sec.tif]

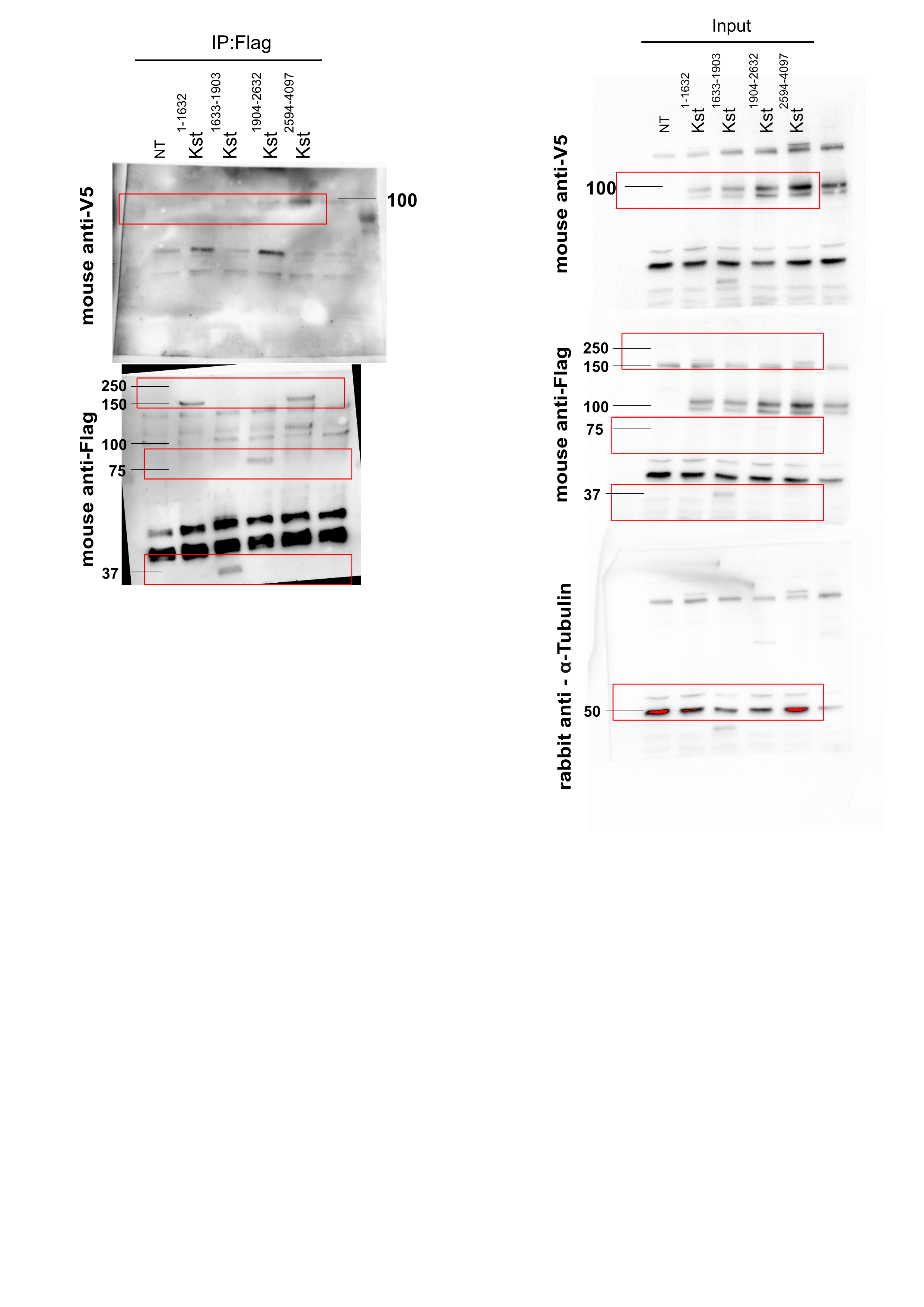

Supplement: Figure 6—source data 1. [file elife-84974-fig6-data1.zip › Figure 6ΓÇösource data 1/raw annd labels 6E.tiff]

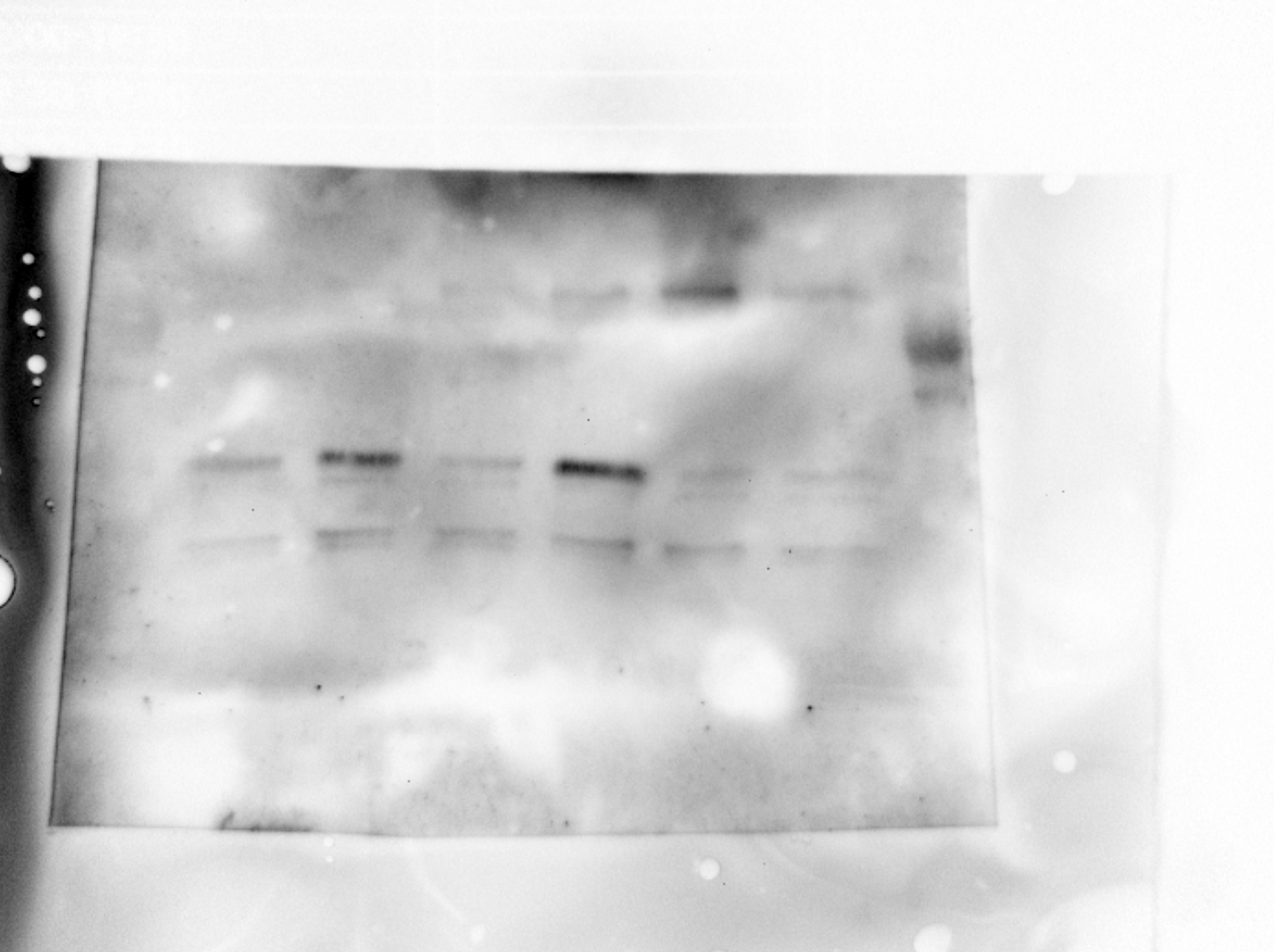

Supplement: Figure 6—source data 1. [file elife-84974-fig6-data1.zip › Figure 6ΓÇösource data 1/raw. IP_ab-v5_60sec.tif]

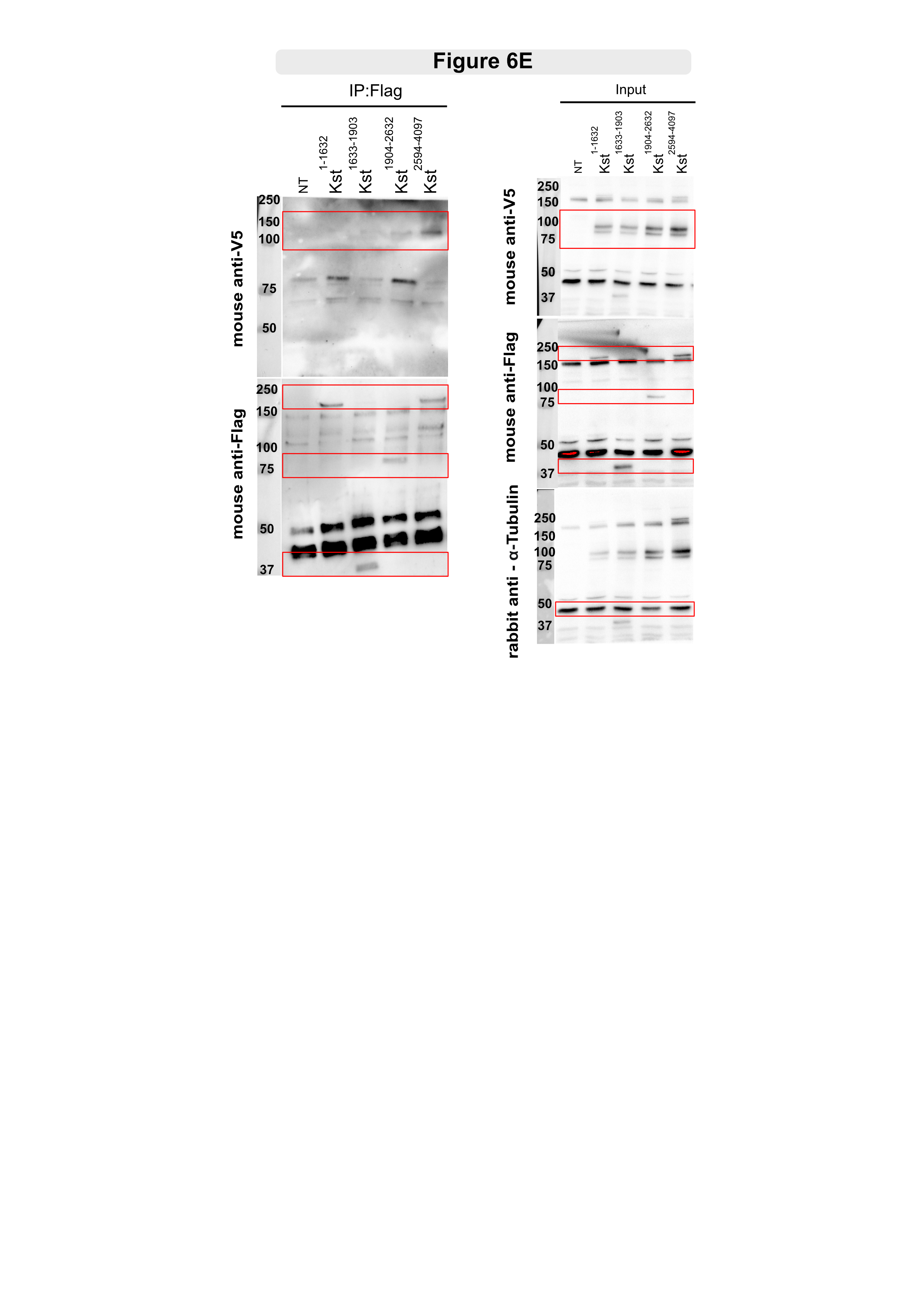

Supplement: Figure 6—source data 1. [file elife-84974-fig6-data1.zip › Figure 6ΓÇösource data 1/Figure 6E.tiff]

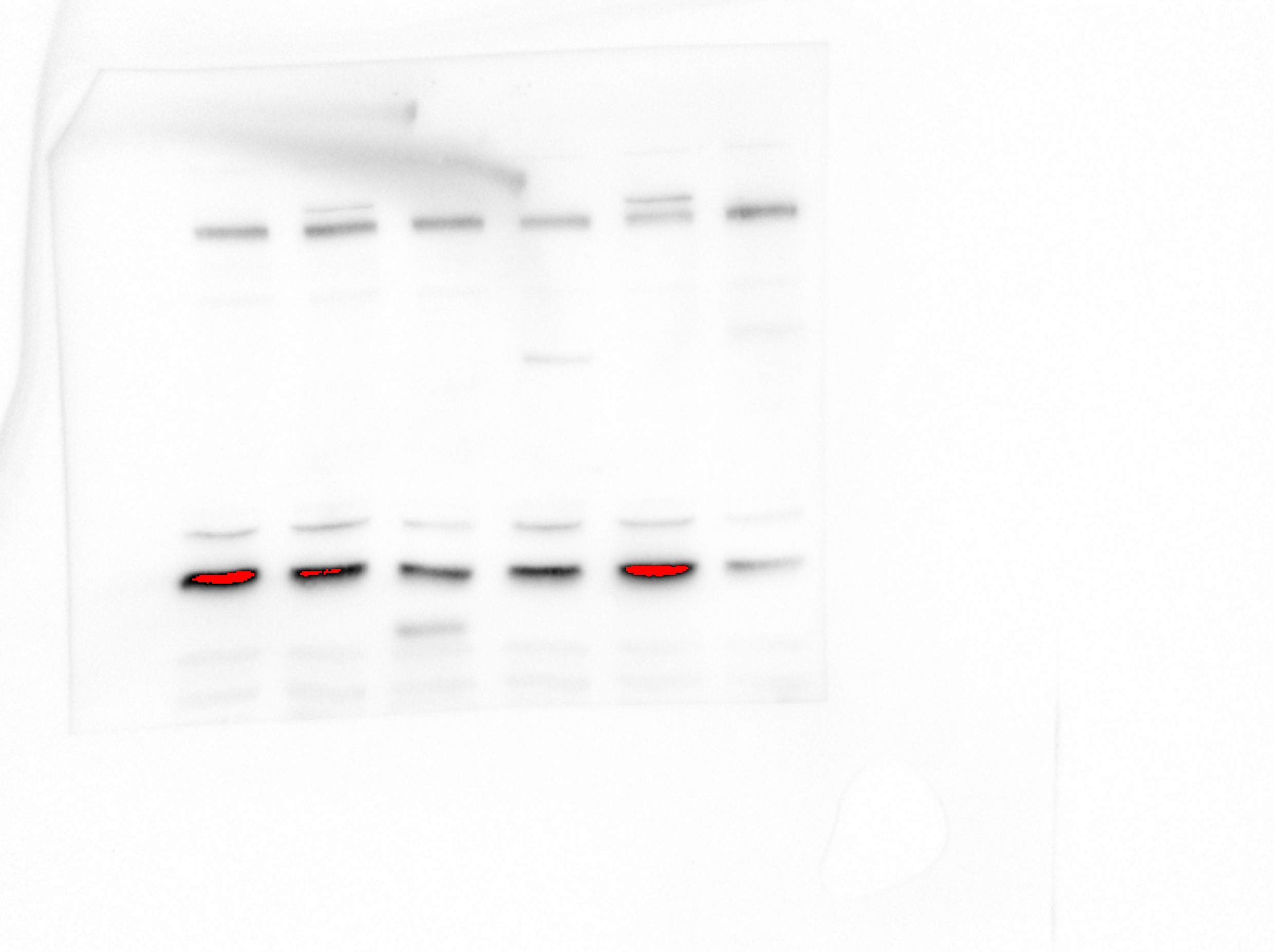

Supplement: Figure 6—source data 1. [file elife-84974-fig6-data1.zip › Figure 6ΓÇösource data 1/raw Input_ab-Flag_5sec.tif]

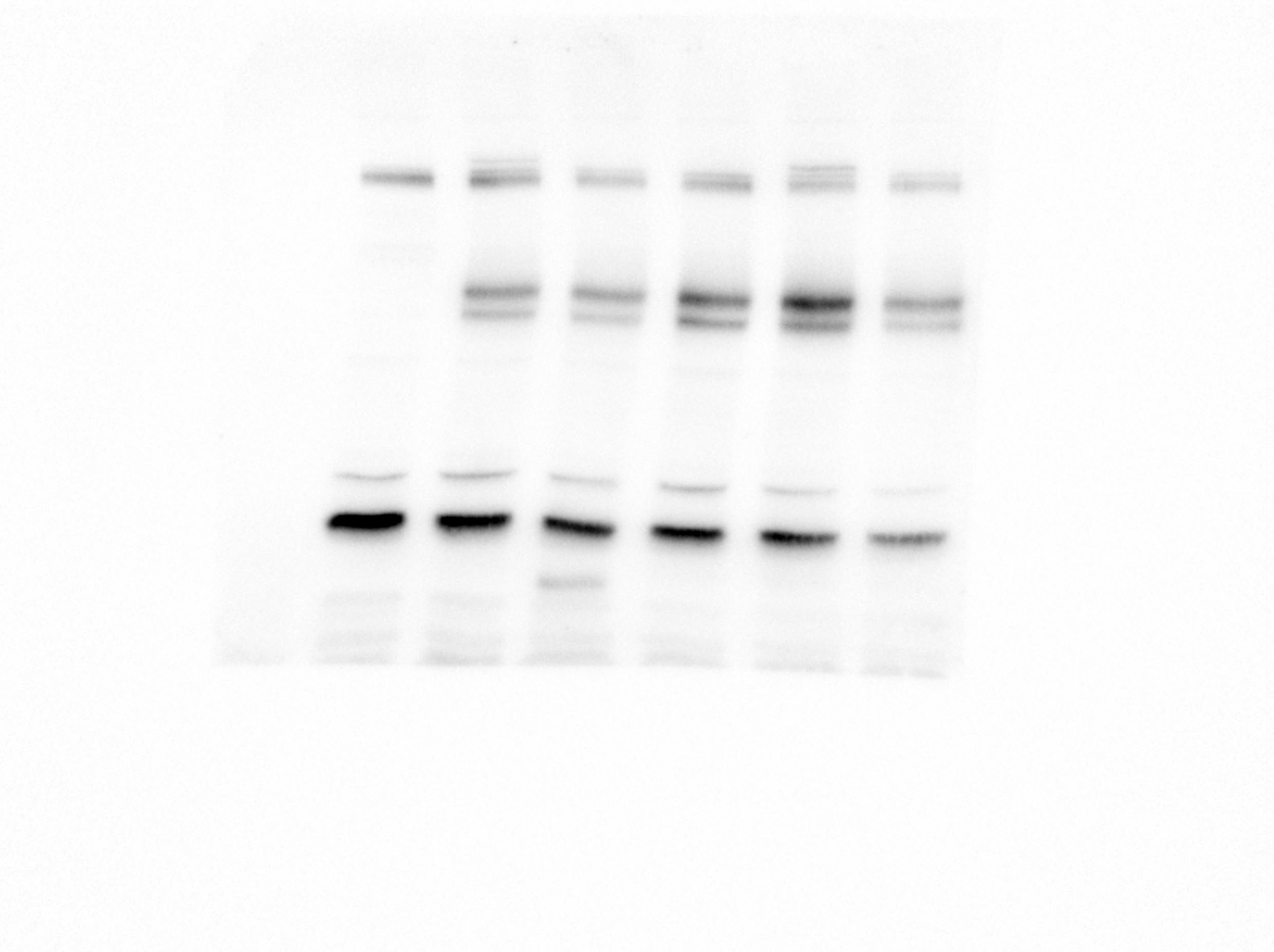

Supplement: Figure 6—source data 1. [file elife-84974-fig6-data1.zip › Figure 6ΓÇösource data 1/raw Input_ab-EMP_5sec.tif]

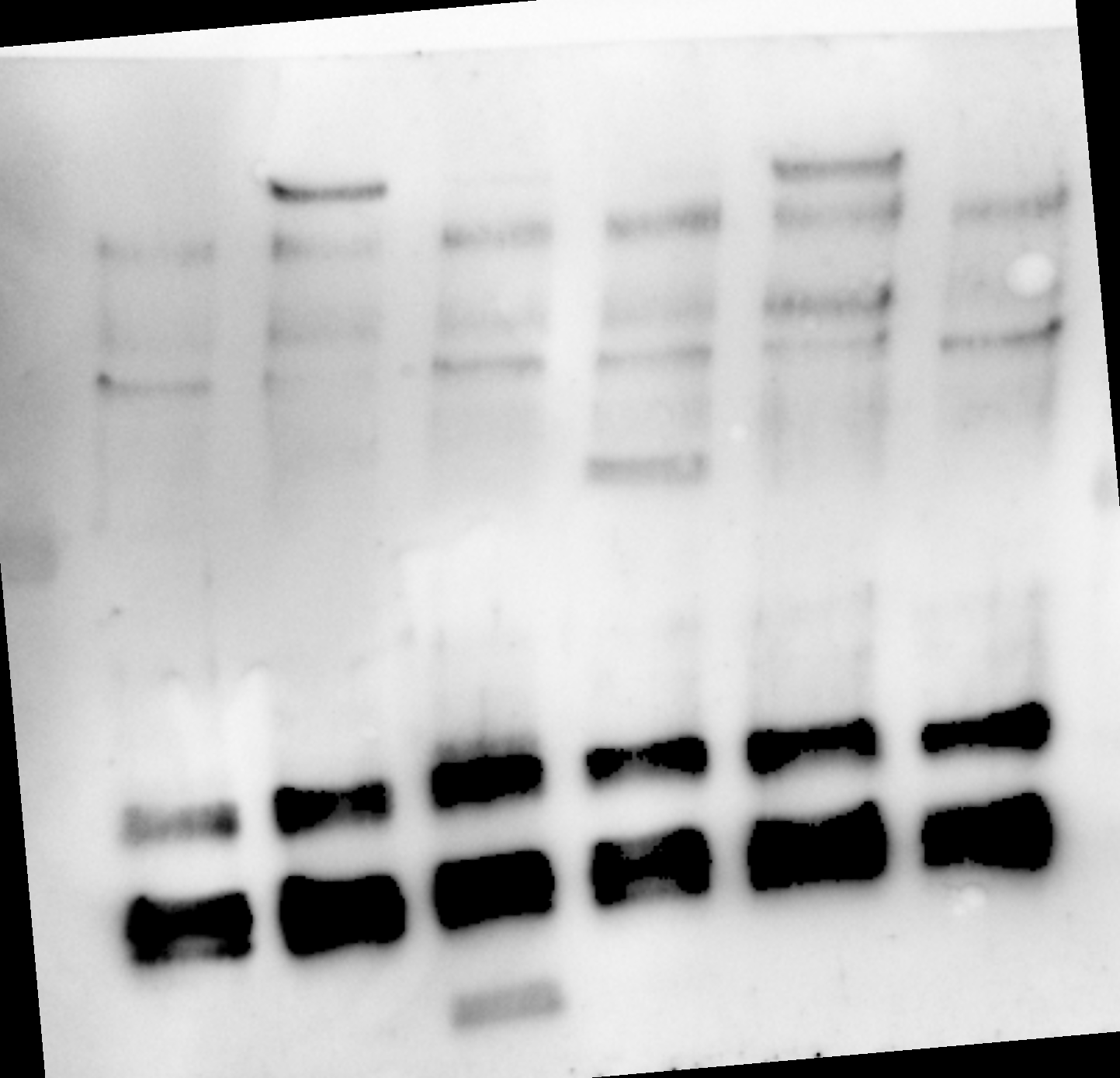

Supplement: Figure 6—source data 1. [file elife-84974-fig6-data1.zip › Figure 6ΓÇösource data 1/raw IP2_ab-Flag_5sec2.tif]

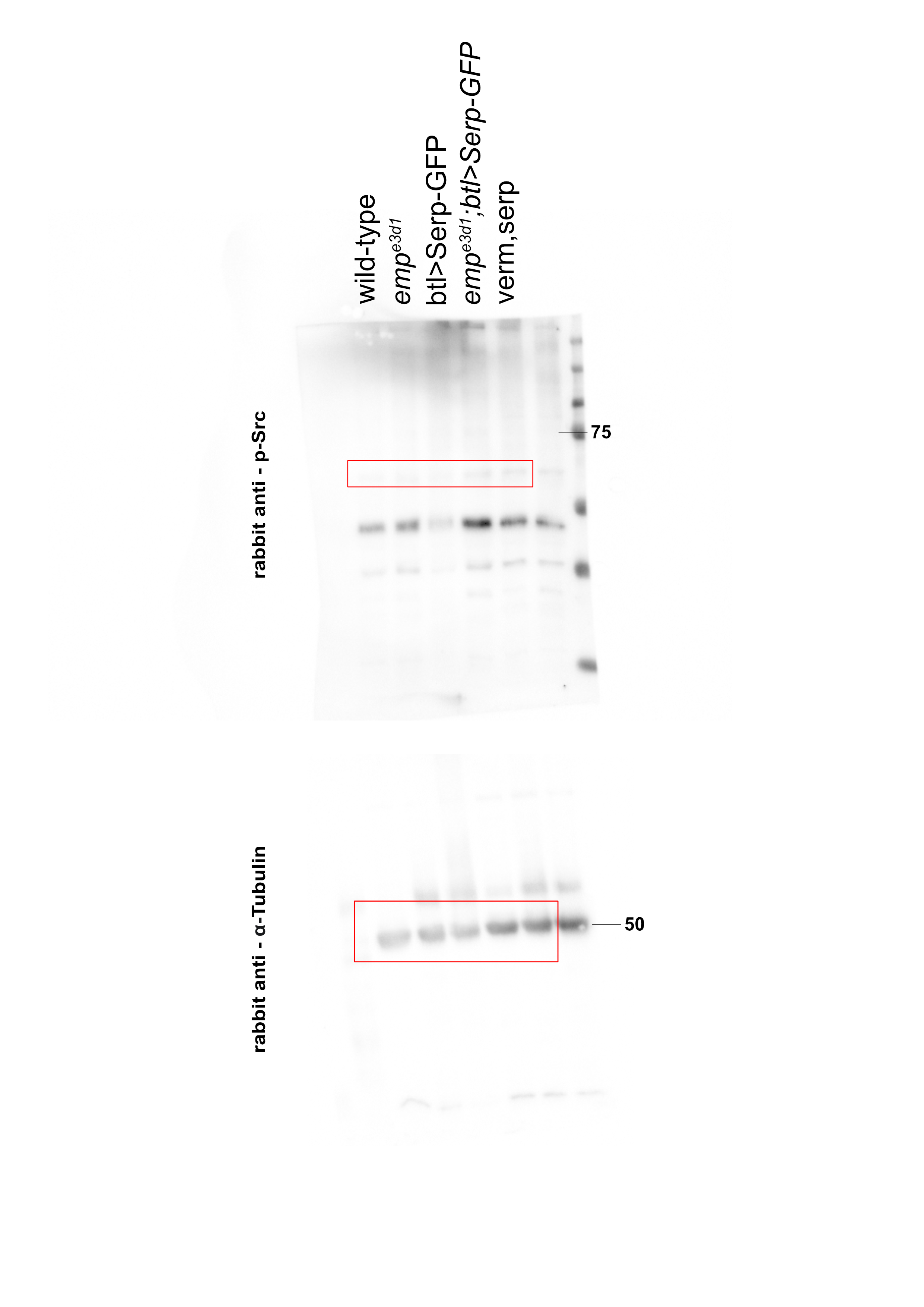

Supplement: Figure 7—source data 1. [file elife-84974-fig7-data1.zip › Figure 7ΓÇösource data 1/raw and labels 7B.tiff]

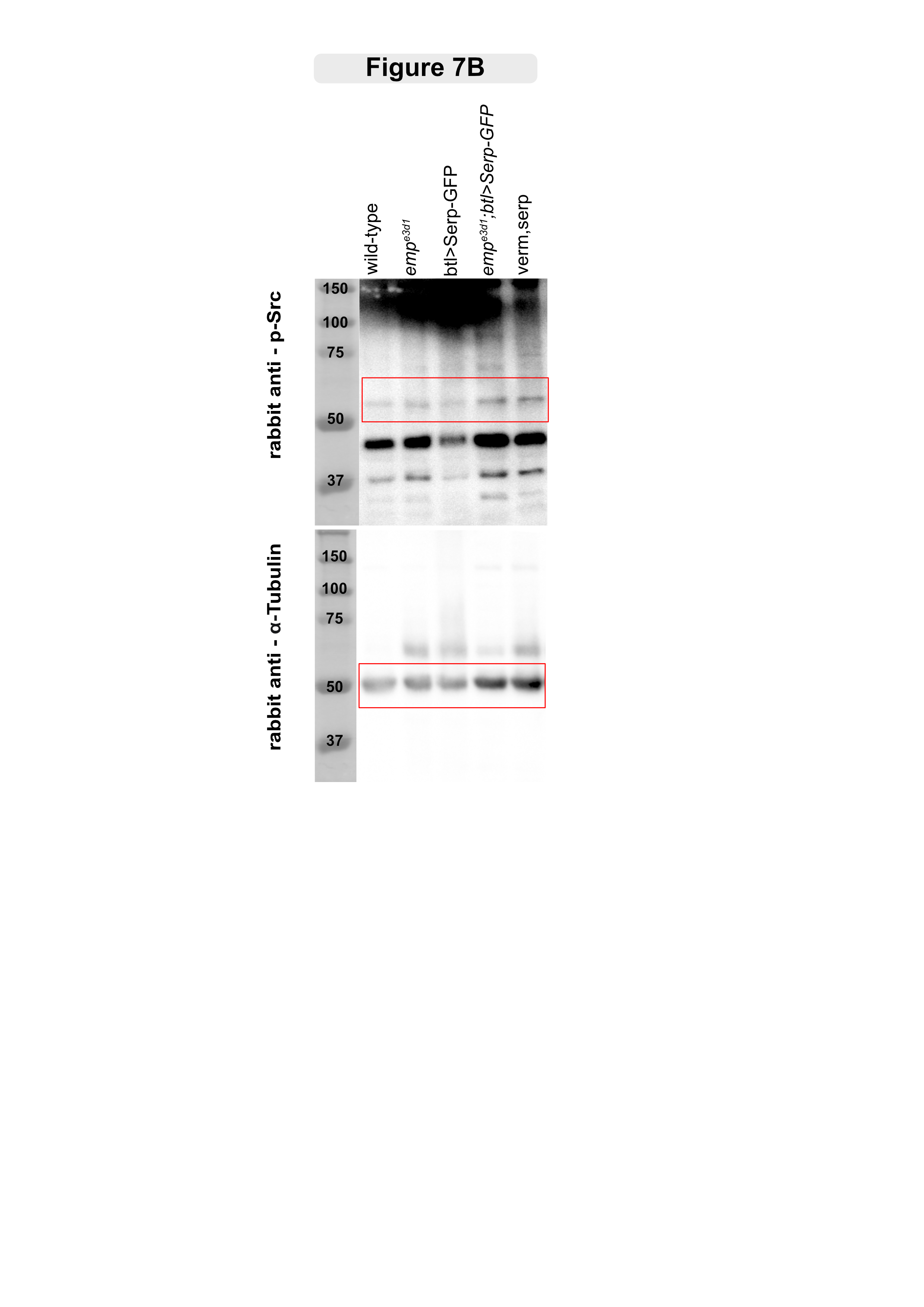

Supplement: Figure 7—source data 1. [file elife-84974-fig7-data1.zip › Figure 7ΓÇösource data 1/Figure 7B.tiff]

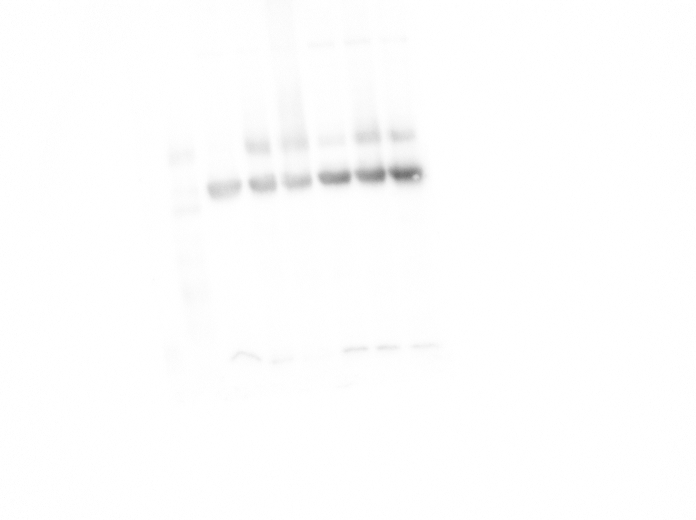

Supplement: Figure 7—source data 1. [file elife-84974-fig7-data1.zip › Figure 7ΓÇösource data 1/raw Memb1_ab-tub_1sec.tif]

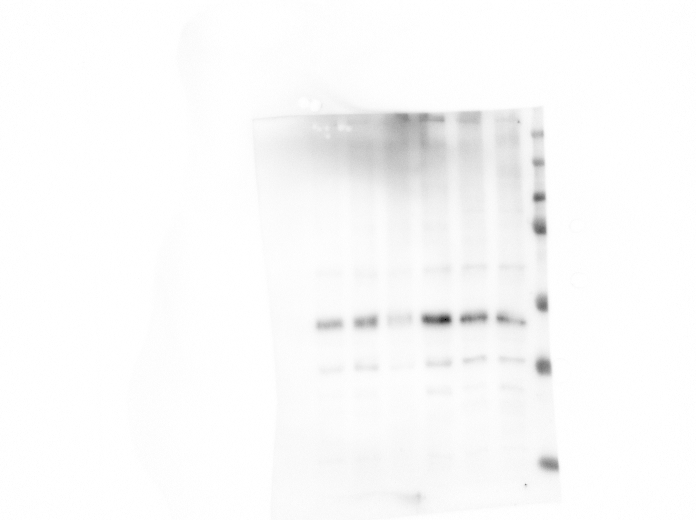

Supplement: Figure 7—source data 1. [file elife-84974-fig7-data1.zip › Figure 7ΓÇösource data 1/raw Memb1_ab-p-Src_10sec.tif]

**Supplementary File 2.**


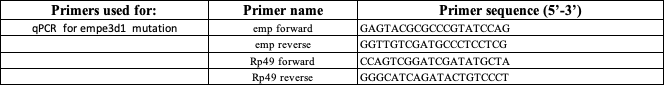

Supplement: Supplementary file 2. [file elife-84974-supp2.docx]
