# Supplementary material for: Scavenger receptor endocytosis controls apical membrane morphogenesis in the Drosophila airways
Source: eLife. 2023 Sep 14;12:e84974. doi: 10.7554/eLife.84974 (PMC10564452; doi:10.7554/eLife.84974)
Supplement: Supplementary file 1. [file elife-84974-supp1.docx]

**Supplementary File 1.**

| ***Drosophila* strains** | **Source** | **Identifier** |
| --- | --- | --- |
| w^1118^ | Bloomington Drosophila Stock | Cat# 3605 |
| *emp^e3d1^/CyOdfdGFPGFP* | This study | N/A |
| *emp^e3d1^/CyOdfdGFP;Dr/TM3Sb,dfdGFP* | This study | N/A |
| *emp^e3d1^/CyOdfdGFP;btlGal4* | This study | N/A |
| *emp^e3d1^/CyOdfdGFP;UAS-Emp/ TM3Sb,dfdGFP* | This study | N/A |
| *emp^e3d1^/CyOdfdGFP;UAS-CD36/ TM3Sb,dfdGFP* | This study | N/A |
| *Btl>Gal4* | (Shiga, Tanaka-Matakatsu and Hayashi, 1996) | N/A |
| *UAS-Gaspmcherry/TM3dfdGFP* | This study | N/A |
| *UAS-SerpGFP/CyOdfdGFP* | (Wang *et al.*, 2006) | N/A |
| *UAS-VermGFP/CyOdfdGFP* | (Wang *et al.*, 2006) | N/A |
| *Chc[1]/FM7c* | Bloomington Drosophila Stock | Cat# 4166 |
| *YFP-Rab5/CyOdfdGFP* | Susanne Eaton | N/A |
| *YFP-Rab7/TM3SbdfdGFP* | Susanne Eaton | N/A |
| *YFP-Rab11/TM3SbdfdGFP* | Susanne Eaton | N/A |
| *UAS-Serp^LDLr^GFP//TM3SbdfdGFP* | (Wang *et al.*, 2006) | N/A |
| *UAS-Serp^CBD^GFP* | (Luschnig *et al.*, 2006) | N/A |
| *UAS-Gasp^LDLr^mcherry/TM3SbdfdGFP* | (Wang *et al.*, 2006) | N/A |
| *UAS-Emp/TM3SbdfdGFP* | This study | N/A |
| *UAS-Emp-GFP/TM3SbdfdGFP* | This study | N/A |
| *verm^ex245^,serp^ex7^/TM3SbdfdGFP* | (Luschnig *et al.*, 2006) | N/A |
| *btl>CAAXmcherry* | Bloomington Drosophila Stock | Cat# 59021 |
| *PBac{681.P.FSVS-1}kst^CPTI002266^* | Kyoto stock center | Cat# [115285](https://kyotofly.kit.jp/cgi-bin/stocks/search_res_det.cgi?DB_NUM=1&DG_NUM=115285) |
| *Kst^2^/TM2LacZ* | (Thomas *et al.*, 1998) | N/A |
| *Src42A^E1^/CyO* | Bloomington Drosophila Stock | Cat# 6408 |
| *emp^e3d1^/CyOdfdGFP; Src42A^E1^/CyOdfdGFP* | This study | N/A |
| [*w^[1118]^;*](http://flybase.org/search/symbol/FBgn/w)*[PBac{681.P.FSVS-1}](http://flybase.org/search/symbol/FBtp/PBac%7B681.P.FSVS-1%7D)*[*kst^[CPTI002266]^*](http://flybase.org/search/symbol/FBti/PBac%7B681.P.FSVS-1%7Dkst%5BCPTI002266%5D) | Kyoto stock center | Cat# [115285](https://kyotofly.kit.jp/cgi-bin/stocks/search_res_det.cgi?DB_NUM=1&DG_NUM=115285) |
| *UAS-moe-GFP* | (Edwards *et al.*, 1997) | N/A |
| *UAS-CD4-Tomato* | Bloomington Drosophila Stock | Cat# 35837 |
| [*Src42A^E1^*](https://flybase.org/reports/FBal0103956)*/CyO;*[*TI{TI}Src64B^ko^*](https://flybase.org/reports/FBti0168556)*/*[*TM6B*](https://flybase.org/reports/FBba0000057)*,*[*Tb^+^*](https://flybase.org/reports/FBal0189742) | Bloomington Drosophila Stock | Cat# 78533 |

*

Edwards, K. A. *et al.* (1997) ‘GFP-moesin illuminates actin cytoskeleton dynamics in living tissue and demonstrates cell shape changes during morphogenesis in Drosophila’, *Developmental Biology*, 191(1), pp. 103–117. doi: 10.1006/dbio.1997.8707.

Luschnig, S. *et al.* (2006) ‘serpentine and vermiform encode matrix proteins with chitin binding and deacetylation domains that limit tracheal tube length in Drosophila’, *Current Biology*, 16(2), pp. 186–194. doi: 10.1016/j.cub.2005.11.072.

Shiga, Y., Tanaka-Matakatsu, M. and Hayashi, S. (1996) ‘A nuclear GFP/beta-galactosidase fusion protein as a marker for morphogenesis in living Drosophila’, *Development, Growth and Differentiation*, 38(1), pp. 99–106. doi: 10.1046/j.1440-169X.1996.00012.x.

Thomas, G. H. *et al.* (1998) ‘Drosophila β(Heavy)-spectrin is essential for development and contributes to specific cell fates in the eye’, *Development*, 125(11), pp. 2125–2134. doi: 10.1242/dev.125.11.2125.

Wang, S. *et al.* (2006) ‘Septate-junction-dependent luminal deposition of chitin deacetylases restricts tube elongation in the Drosophila trachea’, *Current Biology*. doi: 10.1016/j.cub.2005.11.074.
